# Machine Learning Methods for Predicting Long-Term Mortality in Patients After Cardiac Surgery

**DOI:** 10.3389/fcvm.2022.831390

**Published:** 2022-05-03

**Authors:** Yue Yu, Chi Peng, Zhiyuan Zhang, Kejia Shen, Yufeng Zhang, Jian Xiao, Wang Xi, Pei Wang, Jin Rao, Zhichao Jin, Zhinong Wang

**Affiliations:** ^1^Department of Cardiothoracic Surgery, Changzheng Hospital, Naval Medical University, Shanghai, China; ^2^Department of Health Statistics, Naval Medical University, Shanghai, China; ^3^Department of Cardiothoracic Surgery, No. 988 Hospital of Joint Logistic Support Force of PLA, Zhengzhou, China; ^4^Department of Personnel Administration, Second Affiliated Hospital of Naval Medical University, Shanghai, China

**Keywords:** prediction model, machine learning, cardiac surgery, intensive care unit, long-term mortality, MIMIC-III database

## Abstract

**Objective:**

This study aims to construct and validate several machine learning (ML) algorithms to predict long-term mortality and identify risk factors in unselected patients post-cardiac surgery.

**Methods:**

The Medical Information Mart for Intensive Care (MIMIC-III) database was used to perform a retrospective administrative database study. Candidate predictors consisted of the demographics, comorbidity, vital signs, laboratory test results, scoring systems, and treatment information on the first day of ICU admission. Four-year mortality was set as the study outcome. We used the ML methods of logistic regression (LR), artificial neural network (NNET), naïve bayes (NB), gradient boosting machine (GBM), adapting boosting (Ada), random forest (RF), bagged trees (BT), and eXtreme Gradient Boosting (XGB). The prognostic capacity and clinical utility of these ML models were compared using the area under the receiver operating characteristic curves (AUC), calibration curves, and decision curve analysis (DCA).

**Results:**

Of 7,368 patients in MIMIC-III included in the final cohort, a total of 1,337 (18.15%) patients died during a 4-year follow-up. Among 65 variables extracted from the database, a total of 25 predictors were selected using recursive feature elimination and included in the subsequent analysis. The Ada model performed best among eight models in both discriminatory ability with the highest AUC of 0.801 and goodness of fit (visualized by calibration curve). Moreover, the DCA shows that the net benefit of the RF, Ada, and BT models surpassed that of other ML models for almost all threshold probability values. Additionally, through the Ada technique, we determined that red blood cell distribution width (RDW), blood urea nitrogen (BUN), SAPS II, anion gap (AG), age, urine output, chloride, creatinine, congestive heart failure, and SOFA were the Top 10 predictors in the feature importance rankings.

**Conclusions:**

The Ada model performs best in predicting 4-year mortality after cardiac surgery among the eight ML models, which might have significant application in the development of early warning systems for patients following operations.

## Introduction

Every year, two million cardiac surgical procedures are being performed around the world (1). Risk prediction models of patients undergoing cardiac surgery might be helpful for clinicians for alerting, judgment, and intervention to improve postoperative survival (2). Some risk stratifications scores and models have been created to aid clinical decision making such as the original European System for Cardiac Operative Risk Evaluation (EuroSCORE) (3), EuroSCORE II (4), and the North American Society of Thoracic Surgeons (STS) (5–7). The majority of attention in such models has, however, been focused on those that predict short-term outcomes. There has been much less attention paid to the prediction of long-term outcomes, which are probably an equivalent indication of surgeon performance and surgical treatment appropriateness. Additionally, most of the prediction scores, using the traditional logistic regression method, were developed assuming that the predictors interact in a linear and additive way (8), despite the reality that the interactions are often non-linear and multifactorial (9). It might influence the predictive power of these scores. Several studies have reported that some of these scores overestimate the risk of mortality for patients with low risk in actuality while underestimating the risk for high-risk patients (10–15).

Machine learning (ML), a branch of artificial intelligence, is a relatively new technique that arose from the development of complicated algorithms and the analysis of enormous datasets (16). ML has been applied in areas of medicine such as diagnosis, interpretation of medical imaging, treatment strategies, and outcome prediction (17). ML models can provide new insight into complicated interactions, non-linearities, unrecognized patterns and correlations, and the importance of trends in the explanatory variables (18). There are a growing number of studies that ML models could provide a more accurate risk prediction compared to conventional statistical methods. Moreover, several recent studies have applied ML to predict short-term mortality in patients after cardiac surgery (19–21). However, to the best of our knowledge, no predictive model for long-term mortality has been constructed targeting unselected patients post-cardiac surgery using ML techniques.

In the present study, we aimed to construct and validate eight ML models using easily accessible, early-stage, and well-generalized variables to predict long-term mortality and identify risk factors in patients after cardiac surgery during a 4-year follow-up.

## Methods

### Study Design and Data Resource

Based on the methods employed in our previous studies (22–25), we conducted a retrospective analysis using all the relevant data extracted from the Medical Information Mart for Intensive Care (MIMIC-III) database. The MIMIC-III database is an open and publicly available database that contains high-quality data from over 50,000 patients admitted to intensive care units (ICU) at the Beth Israel Deaconess Medical Center (26). After passing the “Protecting Human Research Participants” exam, we were granted access to the dataset (authorization codes: 33281932 and 41657645). Since the study was an analysis of a third-party anonymized publicly available database with pre-existing institutional review board approval, the ethical approval statement and the requirement for informed consent were waived. In summary, this study conformed to the provisions of the Declaration of Helsinki (as revised in Edinburgh 2000). This study was reported according to the transparent reporting of a multivariable prediction model for individual prognosis or diagnosis (TRIPOD) guideline (27).

### Patient Selection

Of all patients in the MIMIC-III database, we included patients as follows: (1) age older than 18 years; (2) those who underwent cardiac surgery including coronary artery bypass grafting (CABG), valvular operation, revision procedures, and some indicators of cardiac surgery. Patients were excluded if they had: (1) multiple ICU admission; (2) a length of stay in the ICU <24 h; and (3) incomplete follow-up information.

### Data Extraction and Processing

Demographics, vital signs, laboratory tests, scoring systems, treatment information, and others were extracted from the MIMIC-III database using structured query language with PostgreSQL (version 9.4.6, www.postgresql.org). Only early-stage clinical and laboratory variables that can be obtained on the first day of ICU admission were incorporated in the prediction model. If patients received vital signs measurement or laboratory tests more than once on the first day of admission, only the initial test results were considered for subsequent analyses. For privacy considerations, the MIMIC-III database changes the date of birth to exactly 300 years before admission for those patients over the age of 89 at the time of admission. As a result, values of 300 for “age” were reverted to 89.

The subject IDs were used to identify distinct adult patients. The predictors included: (1) demographics: age, gender, and ethnicity; (2) comorbidities: coronary artery disease, congestive heart failure, valvular disease, active endocarditis, cardiac arrhythmias, hypertension, pulmonary circulation disorders, chronic pulmonary disease, peripheral vascular disease, stroke, diabetes, dyslipidemia, anemia, renal failure, liver disease, coagulopathy, metastatic cancer, solid tumor (without metastasis), hypothyroidism, fluid and electrolyte disorders, obesity, weight loss, alcohol abuse, drug abuse, and smoker; (3) vital signs: systolic blood pressure (SBP), diastolic blood pressure (DBP), mean blood pressure (MBP), heart rate, respiratory rate, temperature, and urine output; (4) Laboratory findings: white blood cell (WBC), red blood cell (RBC), platelet, red blood cell distribution width (RDW), hematocrit, hemoglobin, sodium, potassium, calcium, magnesium, chloride, phosphate, prothrombin time (PT), international normalized ratio (INR), SpO_2_, pH, base excess (BE), anion gap (AG), bicarbonate, glucose, blood urea nitrogen (BUN), and creatinine; (5) prognostic scoring system: Sequential Organ Failure Assessment (SOFA), quick Sequential Organ Failure Assessment (qSOFA), and Simplified Acute Physiology Score II (SAPS II); (6) Treatment information: surgical type, mechanical ventilation, renal replacement therapy (RRT), and extracorporeal membrane oxygenation (ECMO). Finally, 4-year mortality was set as the study outcome.

### Management of Missing Data

As extensive missing data might lead to bias, variables with over 20% missing values were excluded. Correspondingly, multivariable imputation was applied for variables with fewer than 20% missing values (28). Additionally, the extreme and error values were not omitted and treated as missing data for imputation. Variables for which multivariable imputation was adopted were listed in Supplementary Table 1.

### Statistical Analysis

Values were presented as total numbers with percentages for categorical variables and the means with standard deviations (if normal) or medians with interquartile ranges (IQR) (if non-normal) for continuous variables. Proportions were compared using χ^2^ test or Fisher exact tests while continuous variables were compared using the Student *t*-test, or Wilcoxon rank-sum test, as appropriate.

In this study, the data were divided at random, with 70% utilized for training and 30% for testing. The most relevant variables were selected using recursive feature elimination (RFE) as a feature selection approach. In short, RFE recursively fits a model based on smaller feature sets until a specified termination criterion is reached. In each loop, in the trained model, features are ranked based on their importance. Finally, dependency and collinearity were eliminated. Features were then considered in groups of 5/15/25/35/45/55/ALL (ALL = 65 variables) organized by the ranks obtained after the feature selection method. To find the optimal hyperparameters, 5-fold cross-validation was used as a resampling method. In each iteration, every 9 folds are used as a training subset, and the remaining 1 fold was processed to tune the hyperparameters. This training-testing process was repeated thirty times. And in this way, each sample would be involved in the training model, and also participate in the testing model, so that all data were used to the greatest extent. In this study, we employed multiple diverse ML algorithms to develop models, containing artificial neural network (NNET), naïve bayes (NB), gradient boosting machine (GBM), adapting boosting (Ada), random forest (RF), bagged trees (BT), eXtreme Gradient Boosting (XGB), and logistic regression (LR). Initially, we conducted internal validation on the development sets to quantify optimism in the predictive performance and evaluate the stability of the prediction model. We use the Cross-validation technique with 30 repeats of 5-fold cross-validation to evaluate the internal validity of each model. All the models were assessed in multiple dimensions regarding their model performance. The median and 95% confidence intervals of the area under the receiver operating characteristic curves (AUC) were calculated, where an AUC value of 1.0 means perfect discrimination and 0.5 represents no discrimination. And the accuracy, sensitivity, specificity, negative predictive value, and positive predictive value were also calculated. Calibration plots were drawn to visualize the prediction abilities of the models. To determine the clinical usefulness of the included variables by quantifying the net benefit at different threshold probabilities, we conducted the decision curve analysis (DCA) (19). For the best-performing model, the significance of the model parameters was identified and reported. Finally, the “Shiny” package in the R was used to construct a visual data analysis platform.

All analyses were performed by the statistical software packages R version 4.0.2 (http://www.R-project.org, The R Foundation). In our study, we used the “Caret” R packages to achieve the process. *P* < 0.05 (two-sided test) were considered statistically significant.

## Result

### Baseline Characteristics

**Figure 1:**
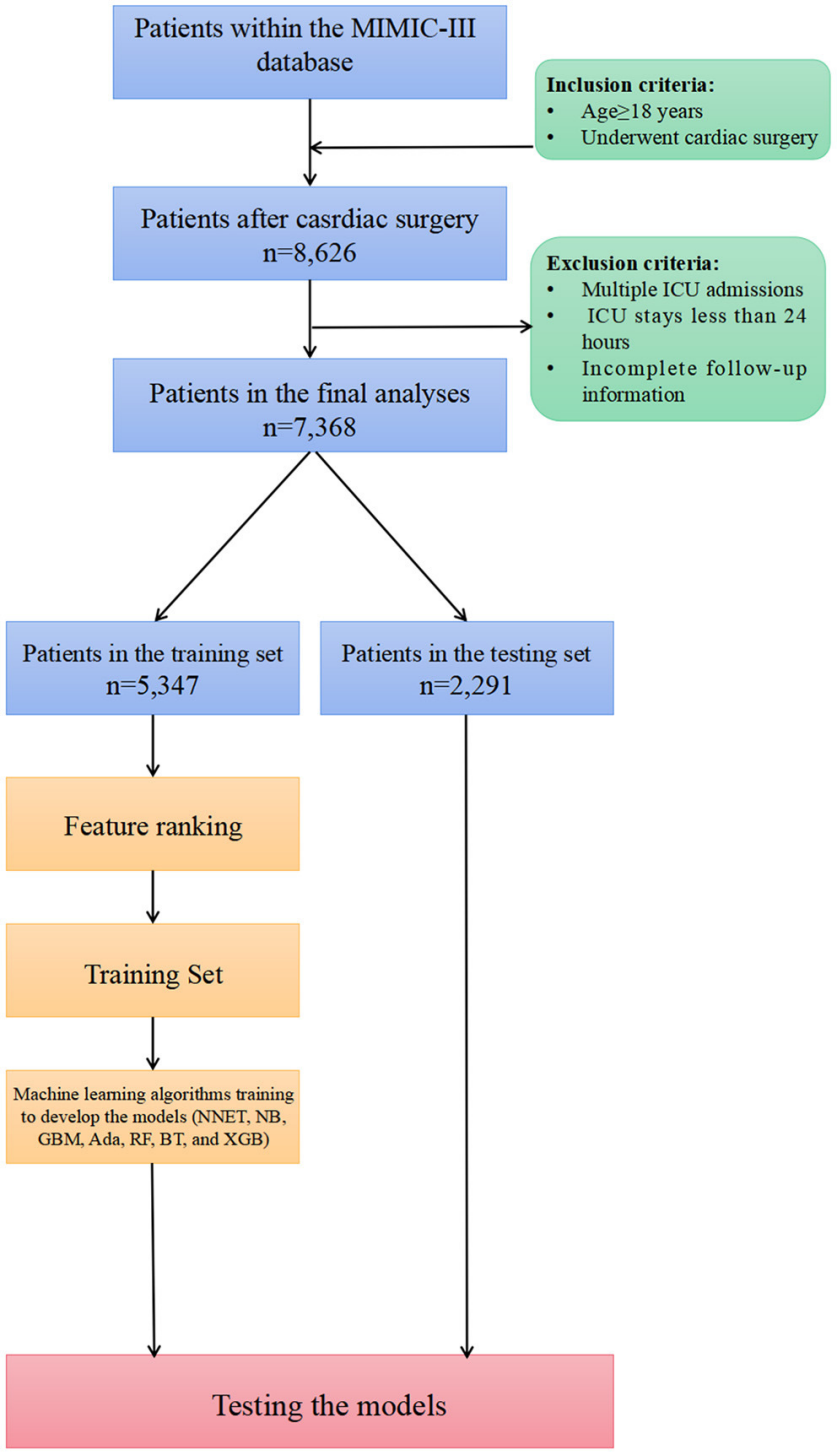
Overview of the methods used for data extraction, training, and testing. MIMIC, Medical Information Mart for Intensive Care; ICU, intensive care units; NNET, artificial neural network; NB, naïve bayes; GBM, gradient boosting machine; Ada, adapting boosting; RF, random forest; BT, bagged trees; XGB, eXtreme Gradient Boosting; LR, logistic regression.

In total, 7,368 patients fulfilled the selection criteria and comprised the final study cohort (Figure 1). The mortality rate of the cohort was 18.15% (6,301 survivors and 1,337 non-survivors) during a 4-year follow-up. The comparison of characteristics between the survivors and the non-survivors is reported in Table 1. Non-survivors were older (*P* < 0.001) and tended to be female (*P* < 0.001) with the medical history of congestive heart failure (*P* < 0.001), valvular disease (*P* = 0.005), active endocarditis (*P* = 0.048), cardiac arrhythmias (*P* < 0.001), pulmonary circulation disorders (*P* < 0.001), chronic pulmonary disease (*P* < 0.001), peripheral vascular disease (*P* < 0.001), stroke (*P* = 0.047), diabetes (*P* = 0.009), renal failure (*P* < 0.001), liver disease (*P* < 0.001), coagulopathy (*P* < 0.001), metastatic cancer (*P* < 0.001), solid tumor (*P* = 0.013), fluid and electrolyte disorders (*P* < 0.001), and weight loss (*P* < 0.001). Regarding vital signs and laboratory findings, non-survivors were more likely to have higher SBP (*P* = 0.014), higher heart rate (*P* = 0.010), higher respiratory rate (*P* = 0.009), lower temperature (*P* < 0.001), lower urine output (*P* < 0.001), higher anion gap (*P* < 0.001), lower RBC (*P* = 0.010), higher platelet (*P* < 0.001), higher RDW (*P* < 0.001), lower hemoglobin (*P* < 0.001), BUN (*P* < 0.001), higher creatinine (*P* < 0.001), higher calcium (*P* < 0.001), higher potassium (*P* < 0.001), lower sodium (*P* < 0.001), higher phosphate (*P* < 0.001), lower chloride (*P* < 0.001), higher SOFA (*P* < 0.001), and higher SAPS II (*P* < 0.001). Moreover, patients who died during follow-up were also more likely to receive RRT (*P* < 0.001) and ECMO (*P* < 0.001).

**Table 1:** Baseline characteristics between survivors and non-survivors.

**Characteristics**	**Survivors (*n* = 6,301)**	**Non-survivors (*n* = 1,337)**	***P*-value**
**Demographics**			
Age, year, median (IQR)	67.00 (58.00, 75.00)	75.00 (65.00, 81.00)	<0.001
Gender, male, *n* (%)	4,337 (68.83)	811 (60.66)	<0.001
Ethnicity, white, *n* (%)	4,551 (72.23)	933 (69.78)	0.077
**Admission type**, ***n*** **(%)**			<0.001
Elective	2,886 (45.80)	367 (27.45)	
Emergency	3,176 (50.40)	901 (67.39)	
Urgent	239 (3.79)	69 (5.16)	
**Comorbidities**, ***n*** **(%)**			
Coronary artery disease	4,402 (69.86)	885 (66.19)	0.009
Congestive heart failure	1,611 (25.57)	673 (50.34)	<0.001
Valvular disease	2,760 (43.80)	643 (48.09)	0.005
Active endocarditis	103 (1.63)	33 (2.47)	0.048
Cardiac arrhythmias	2,772 (43.99)	794 (59.39)	<0.001
Hypertension	4,384 (69.58)	827 (61.85)	<0.001
Pulmonary circulation disorders	445 (7.06)	161 (12.04)	<0.001
Chronic pulmonary disease	898 (14.25)	296 (22.14)	<0.001
Peripheral vascular disease	1,055 (16.74)	283 (21.17)	<0.001
Stroke	441 (7.00)	115 (8.60)	0.047
Diabetes	1,909 (30.30)	454 (33.96)	0.009
Dyslipidemia	1,981 (31.44)	229 (17.13)	<0.001
Anemia	1,069 (16.97)	245 (18.32)	0.248
Renal failure	488 (7.74)	258 (19.30)	<0.001
Liver disease	109 (1.73)	109 (1.73)	<0.001
Coagulopathy	464 (7.36)	175 (13.09)	<0.001
Metastatic cancer	20 (0.32)	88 (6.58)	<0.001
Solid tumor (without metastasis)	75 (1.19)	28 (2.09)	0.013
Hypothyroidism	553 (8.78)	118 (8.83)	0.996
Fluid and electrolyte disorders	711 (11.28)	291 (21.77)	<0.001
Obesity	518 (8.22)	70 (5.24)	<0.001
Weight loss	40 (0.63)	30 (2.24)	<0.001
Alcohol abuse	147 (2.33)	33 (2.47)	0.844
Drug abuse	57 (0.90)	11 (0.82)	0.897
Smoker	3419 (54.26)	751 (56.17)	0.214
**Vital signs, median (IQR)**			
SBP, mmHg	143.00 (133.00,154.00)	145.00 (132.00,158.00)	0.014
DBP, mmHg	75.00 (69.00,82.00)	75.00 (67.00,83.00)	0.261
MBP, mmHg	97.00 (90.00,105.00)	97.00 (89.00,107.00)	0.491
Heat rate, beats/min	97.00 (90.00,108.00)	99.00 (90.00,111.00)	0.010
Respiratory rate, beats/min	26.00 (23.00,30.00)	27.00 (24.00,31.00)	0.009
Temperature, °C	37.70 (37.20,38.00)	37.50 (37.10,38.00)	<0.001
Urine output, ml	2100.00 (1520.00,2870.00)	1626.00 (1040.00,2457.00)	<0.001
**Laboratory findings, median (IQR)**			
WBC, 10^9^/L	13.40 (10.70,16.80)	13.00 (9.70,16.90)	0.001
RBC, 10^9^/L	3.80 (3.21,4.35)	3.71 (3.26,4.21)	0.010
Platelet, 10^9^/L	191.00 (147.50,241.00)	204.00 (156.00,255.00)	<0.001
RDW, %	13.60 (13.10,14.30)	14.20 (13.50,15.00)	<0.001
Hematocrit, %	33.80 (28.60,38.40)	33.35 (29.20,37.40)	0.156
Hemoglobin, g/dL	11.50 (9.80,13.20)	11.20 (9.80,12.62)	<0.001
BUN, mg/dL	17.00 (13.00,21.00)	20.00 (15.00,27.00)	<0.001
Creatinine, mg/dL	0.90 (0.70,1.10)	1.00 (0.80,1.30)	<0.001
Glucose, mg/dL	176.00 (155.00,200.00)	177.00 (149.00,205.00)	0.765
Calcium, mmol/L	8.60 (8.10,9.10)	8.70 (8.20,9.10)	<0.001
Potassium, mmol/L	4.20 (3.90,4.50)	4.30 (3.90,4.60)	<0.001
Sodium, mmol/L	139.00 (137.00,141.00)	138.00 (136.00,141.00)	<0.001
Chloride, mmol/L	106.00 (103.00,110.00)	103.00 (100.00,107.00)	<0.001
Magnesium, mmol/L	2.00 (1.90,2.20)	2.00 (1.80,2.30)	0.348
Phosphate, mmol/L	3.40 (2.90,3.90)	3.60 (3.00,4.10)	<0.001
PT, s	13.80 (12.80,15.20)	13.80 (12.90,15.10)	0.673
INR, s	1.20 (1.10,1.40)	1.20 (1.10,1.40)	0.092
SpO^2^, %	100.00 (100.00,100.00)	100.00 (100.00,100.00)	<0.001
pH	7.41 (7.38,7.44)	7.41 (7.37,7.44)	0.876
BE, mmol/L	1.00 (0.00,3.00)	1.00 (0.00,3.00)	0.082
AG, mmol/L	13.00 (11.00,14.00)	14.00 (12.00,16.00)	<0.001
Bicarbonate, mmol/L	25.00 (23.00,27.00)	25.00 (22.00,27.00)	0.365
**Prognostic scoring system, median (IQR)**			
SOFA	4.00 (3.00,6.00)	5.00 (3.00,7.00)	<0.001
qSOFA	2.00 (2.00,2.00)	2.00 (2.00,2.00)	0.077
SAPS II	32.00 (26.00,40.00)	38.00 (31.00,47.00)	<0.001
Surgical type, CABG, n (%)	3919 (62.20)	698 (52.21)	<0.001
**Treatment information, n (%)**			
Mechanical ventilation	5433 (86.22)	964 (72.10)	<0.001
RRT	61 (0.97)	70 (5.24)	<0.001
ECMO	8 (0.13)	25 (1.87)	<0.001

*CABG, coronary artery bypass grafting; SBP, systolic blood pressure; DBP, diastolic blood pressure; MBP, mean blood pressure; WBC, white blood cell; RBC, red blood cell; RDW, red blood cell distribution width; PT, prothrombin time; INR, international normalized ratio; BE, base excess; AG, anion gap; BUN, blood urea nitrogen; SOFA, Sequential Organ Failure Assessment; qSOFA, quick Sequential Organ Failure Assessment; SAPS II, Simplified Acute Physiology Score II; RRT, renal replacement therapy; ECMO, extracorporeal membrane oxygenation; IQR, interquartile ranges*.

### Variable Importance

**Figure 2:**
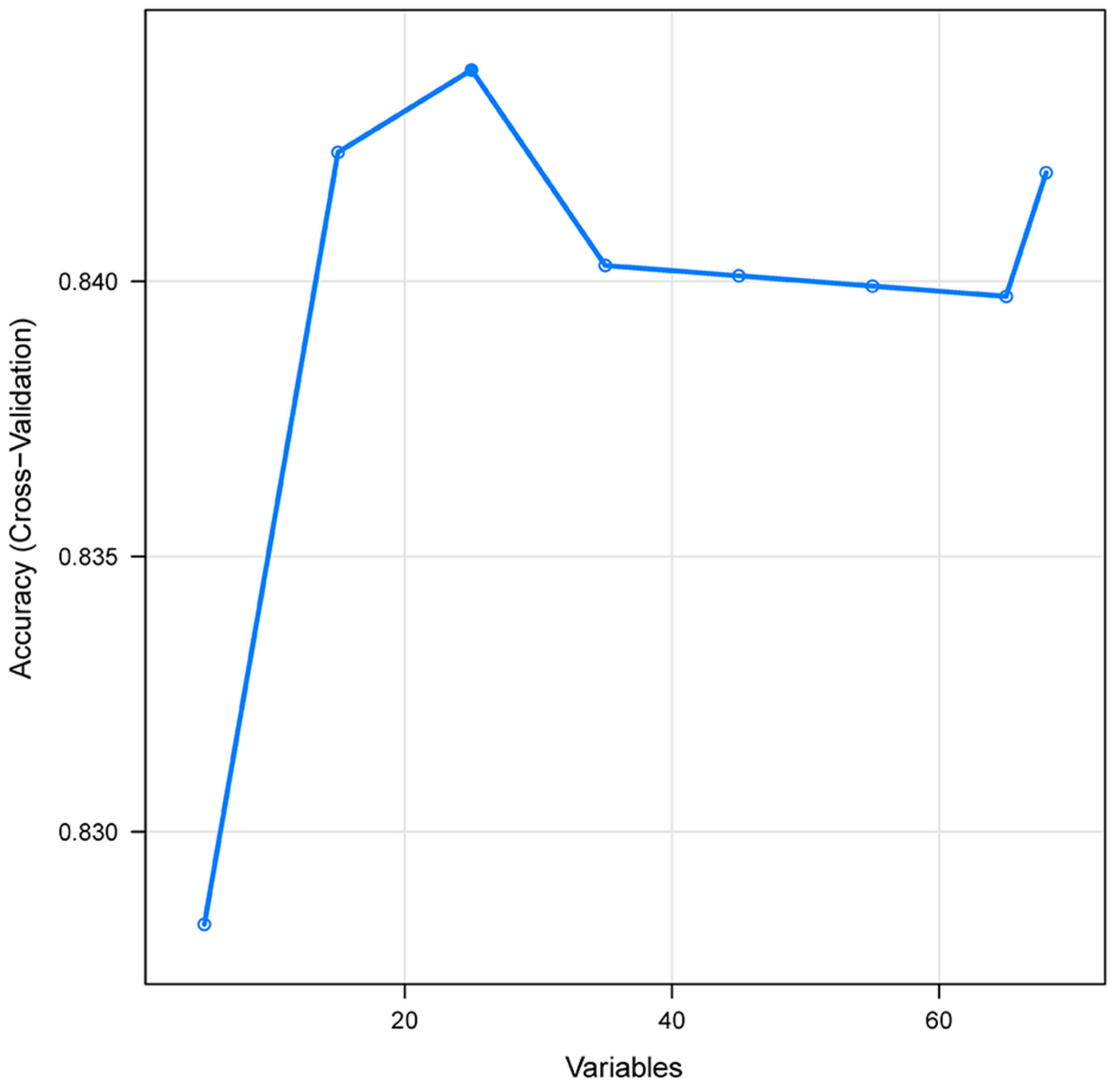
Association between the numbers of variables allowed to be considered at each split and the prediction accuracy in the REF algorithm. REF, recursive feature elimination.

**Figure 3:**
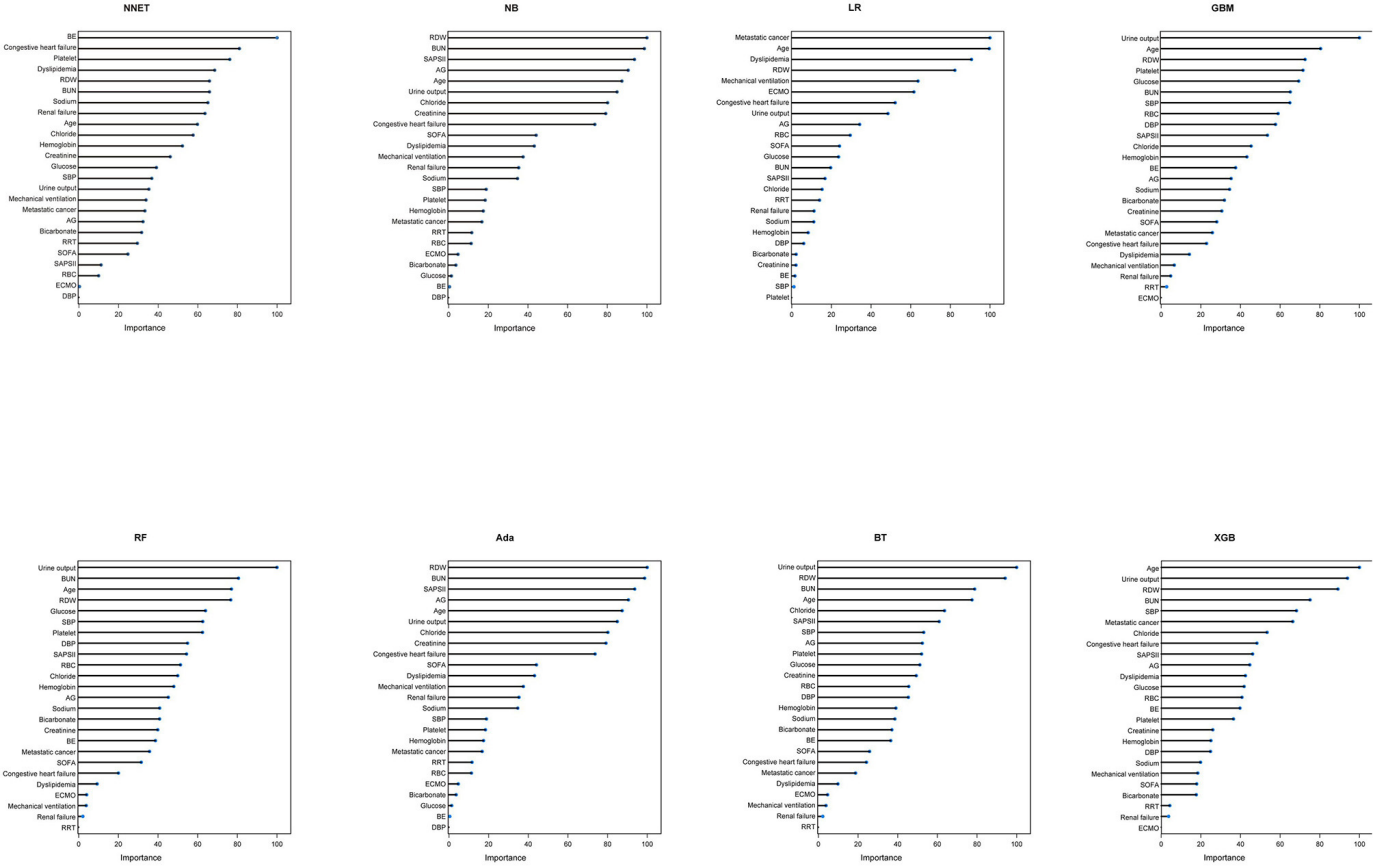
Variable importance in different models. NNET, artificial neural network; NB, naïve bayes; GBM, gradient boosting machine; Ada, adapting boosting; RF, random forest; BT, bagged trees; XGB, eXtreme Gradient Boosting; LR, logistic regression; RDW, red blood cell distribution width; BUN, blood urea nitrogen; SAPS II, Simplified Acute Physiology Score II; AG, anion gap; SOFA, Sequential Organ Failure Assessment; SBP, systolic blood pressure; RRT, renal replacement therapy; RBC, red blood cell; ECMO, extracorporeal membrane oxygenation; BE, base excess; DBP, diastolic blood pressure.

A total of 65 predictors were extracted from the database. Finally, 25 important predictors were selected by the RFE algorithm, including metastatic cancer, urine output, ECMO, RDW, AG, congestive heart failure, mechanical ventilation, sodium, SBP, bicarbonate, DBP, RBC, hemoglobin, age, BUN, chloride, SAPS II, creatinine, RRT, BE, renal failure, dyslipidemia, platelet, SOFA, and glucose (Figure 2). Then, these variables were used in all the subsequent analyses for all models in both training and testing sets. Each variable included in the study had varying importance over 4-year mortality relying on the ML approach (Figure 3). In the Ada model, we determined that RDW, BUN, SAPS II, AG, age, urine output, chloride, creatinine, congestive heart failure, and SOFA were the Top 10 predictors in the feature importance rankings.

### Evaluation of Model Performance

**Figure 4:**
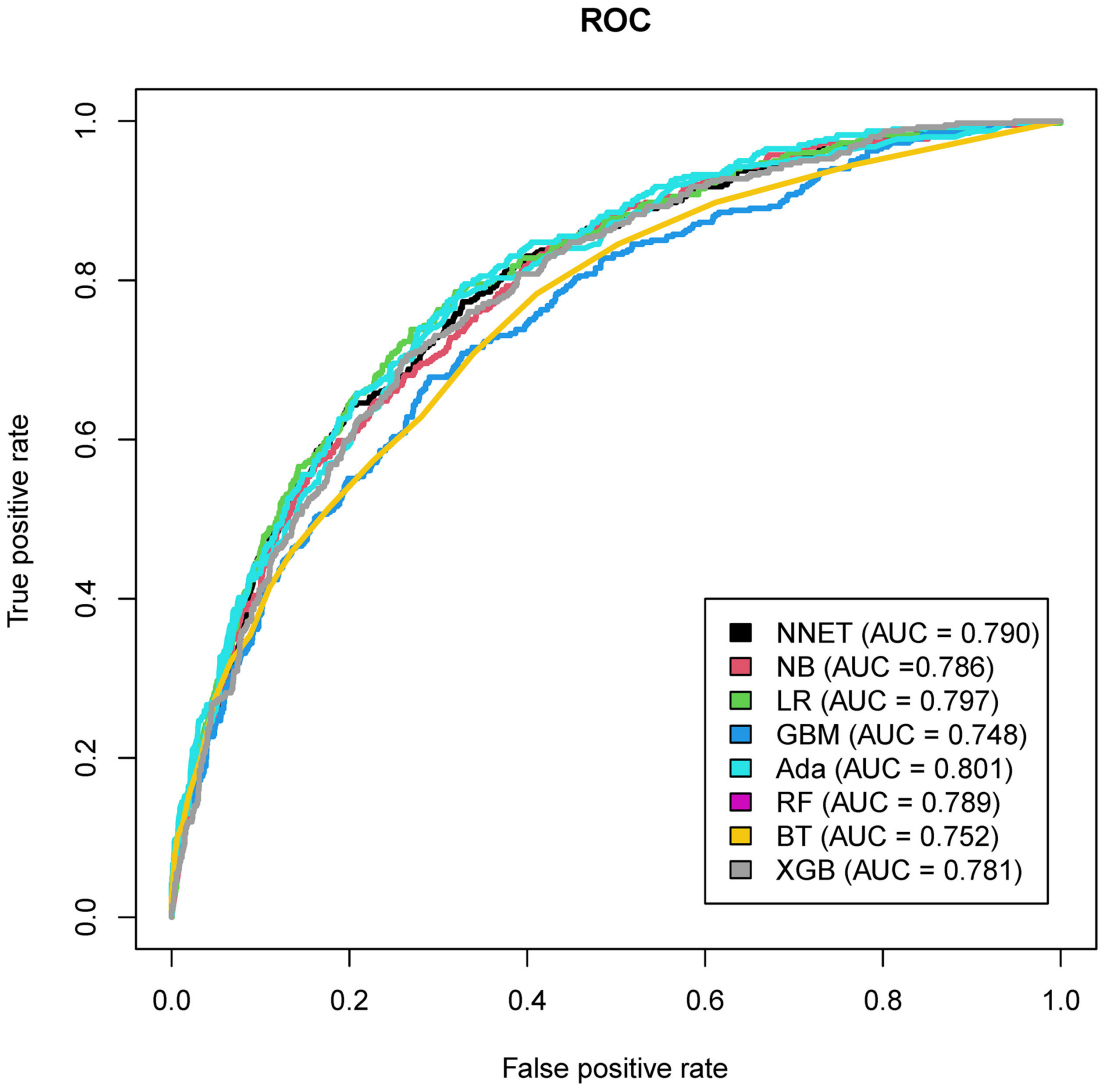
Area under the curve of receiver operating characteristic curve by machine learning models in the validation cohort. ROC, receiver operate characteristics; NNET, artificial neural network; NB, naïve bayes; GBM, gradient boosting machine; Ada, adapting boosting; RF, random forest; BT, bagged trees; XGB, eXtreme Gradient Boosting; LR, logistic regression.

**Figure 5:**
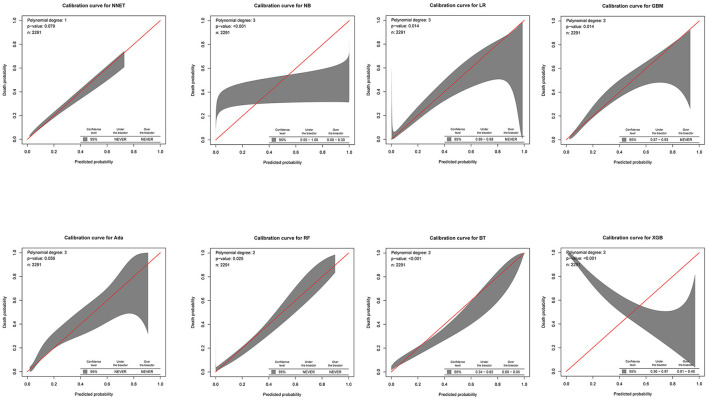
Calibration plots of the eight models. NNET, artificial neural network; NB, naïve bayes; GBM, gradient boosting machine; Ada, adapting boosting; RF, random forest; BT, bagged trees; XGB, eXtreme Gradient Boosting; LR, logistic regression.

**Figure 6:**
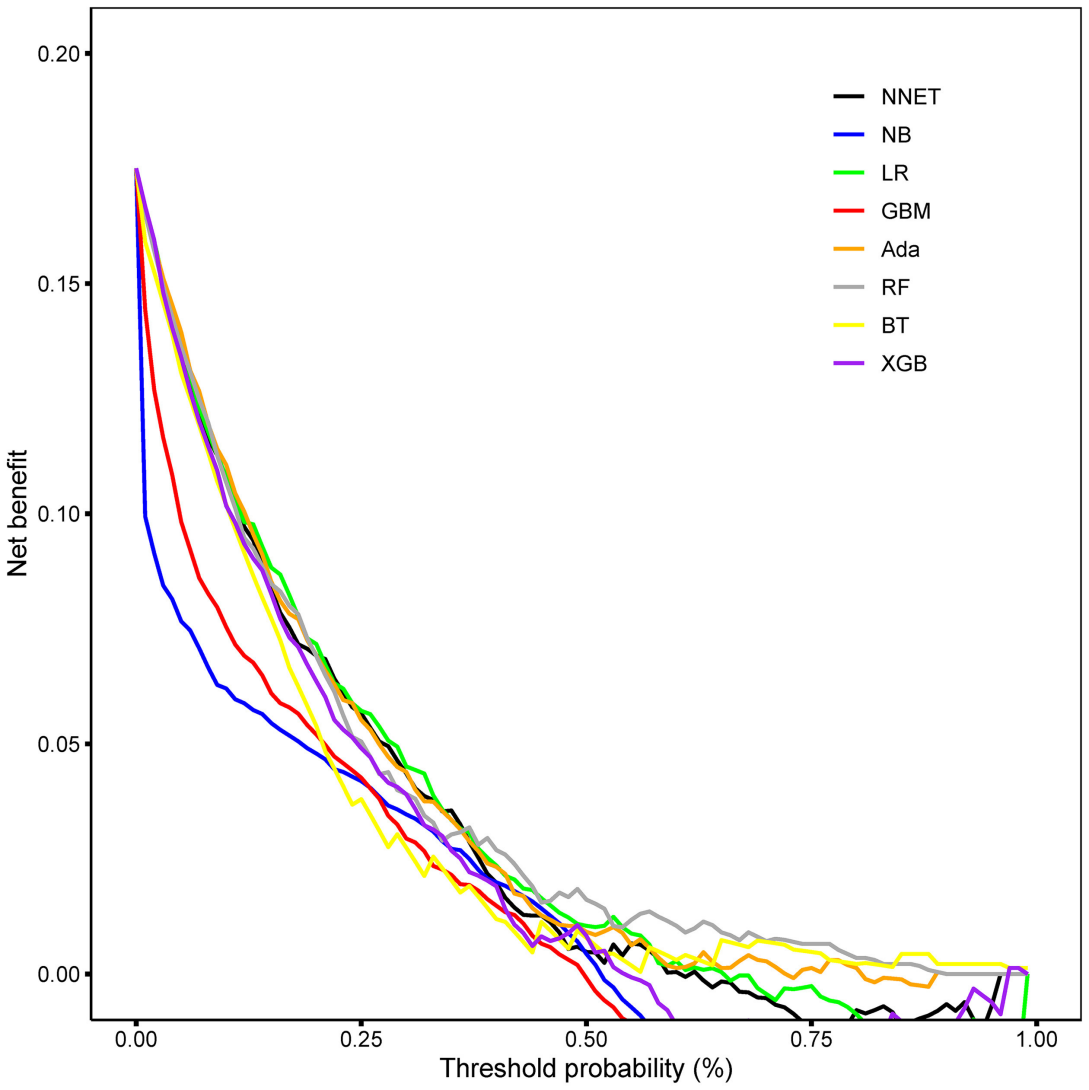
Decision curve analysis of the eight models. NNET, artificial neural network; NB, naïve bayes; GBM, gradient boosting machine; Ada, adapting boosting; RF, random forest; BT, bagged trees; XGB, eXtreme Gradient Boosting; LR, logistic regression.

The discriminatory abilities of all models for the prediction of mortality are in Figure 4, Table 2. Within the training set, the NNET, NB, LR, GBM, Ada, RF, BT, and XGB models were established, and the testing set obtained AUCs of 0.790, 0.786, 0.797, 0.748, 0.801, 0.789, 0.752, and 0.781, respectively. Comparatively, the Ada model had the highest predictive performance among these eight models (AUC 0.801, 95% CI: 0.784–0.817). Calibration plots of the eight models are presented in Figure 5. The calibration curves of NNET and Ada performed better than the other models. The decision curve compared the net benefit of the best model and alternative approaches for clinical decision making. As is shown in Figure 6, the net benefit of the RF, Ada, and BT models surpassed that of other ML models for almost all threshold values, showing that these three models were more superior in predicting the risk of 4-year deaths in this cohort.

**Table 2:** Prediction performance of the machine learning models in the test set.

**Model**	**Accuracy**	**Sensitivity**	**Specificity**	**PPV**	**NPV**	**AUC**	**95%CI**
NNET	0.830	0.773	0.673	0.334	0.933	0.790	(0.772–0.806)
NB	0.829	0.825	0.596	0.302	0.941	0.786	(0.768–0.802)
LR	0.835	0.738	0.731	0.368	0.929	0.797	(0.780–0.814)
GBM	0.824	0.678	0.710	0.332	0.912	0.748	(0.729–0.765)
Ada	0.834	0.793	0.673	0.340	0.938	0.801	(0.784–0.817)
RF	0.841	0.781	0.677	0.339	0.938	0.789	(0.772–0.806)
BT	0.833	0.783	0.589	0.288	0.928	0.752	(0.734–0.770)
XGB	0.833	0.706	0.735	0.361	0.922	0.781	(0.763–0.798)

*NNET, artificial neural network; NB, naïve bayes; GBM, gradient boosting machine; Ada, adapting boosting; RF, random forest; BT, bagged trees; XGB, eXtreme Gradient Boosting; LR, logistic regression; AUC, area under the curve; PPV, positive predictive values; NPV, negative predictive values, AUC, area under the curve; CI, confifidence interval*.

### Development of Webservers for Convenient Clinical Use

**Figure 7:**
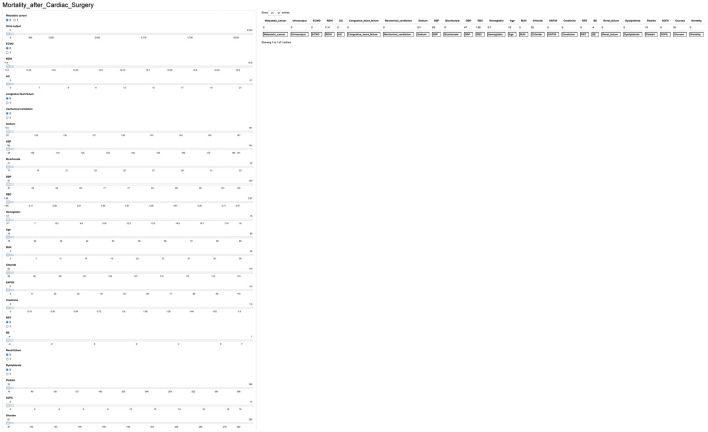
Interface of website usage. RDW, red blood cell distribution width; BUN, blood urea nitrogen; SAPS II, Simplified Acute Physiology Score II; AG, anion gap; SOFA, Sequential Organ Failure Assessment; SBP, systolic blood pressure; RRT, renal replacement therapy; RBC, red blood cell; ECMO, extracorporeal membrane oxygenation; BE, base excess; DBP, diastolic blood pressure.

We next used the Shiny to illustrate the impacts of key features on the death prediction model in individual patients. One visualized and publicly accessible online calculator based on the Ada model was built (https://pengchi2009.shinyapps.io/cardic/) (Figure 7). The webservers may generate an estimated survival probability by entering the covariates.

## Discussion

Long-term mortality risk prediction tools for cardiac surgery can play an important role in enhancing continuity of care and planning resource allocation appropriately. With the advancement of electronic medical records and artificial intelligence, ML algorithms have become more widely utilized in individualized medicine to assist clinical decision-making (29). In this study, several ML algorithms (NNET, NB, GBM, Ada, RF, BT, LR, and XGB) were developed and validated to predict 4-year mortality of patients undergoing cardiac surgery. Concerning the predictive performance, the Ada model exhibited the greatest AUC and outperformed the remaining ML models. Moreover, to help surgeons use the model, a visualized and publicly accessible online calculator was developed, which provided a user-friendly interface. This study was the first to establish a long-term prediction model after cardiac surgery using early-stage and easily obtained variables based on ML methods. It is worth noting that early and accurate prediction of long-term mortality in patients post-cardiac surgery could provide more time for clinicians to offer individualized treatment strategies.

Cardiac surgery, as a unique operation type, had a significant impact on circulation and physiology, as well as posing significant hurdles in terms of lowering mortality (30). In the field of cardiac surgery, there has been an increasing interest in risk prediction models for clinical use. Various risk stratification methods were cited in European guidelines for decision making, even though these scores cannot replace clinical judgment and multidisciplinary dialogue (31). Among the many scores that have been proposed, the original EuroSCORE, EuroSCORE II and STS scores are the most widely used to predict mortality after cardiac surgery. However, several studies have reported that these scores have limitations in some surgeries or patient subgroups (11–13). Recently, a growing number of studies have focused on mid-term or long-term mortality after cardiac surgery (32–36). For example, Wu et al. (37) created a risk score predicting long-term mortality following isolated CABG surgery with the C-statistics ranging from 0.768 to 0.783 for mortality at 1, 3, 5, and 7 years of follow-up. Due to the need for more precise prediction models, the application of ML approaches has been increasingly studied. A recent meta-analysis using 15 studies showed that when compared with LR, ML models provide better discrimination in operative mortality prediction after cardiac surgery (38). In the present study, the Ada model had a better performance in both discriminatory ability with the higher AUC of 0.804 and goodness of fit (visualized by calibration curve) compared to the traditional LR methods.

The potential advantage of ML models is their capacity to capture nonlinearity and the interactions among features without the need for the modeler to manually specify all interactions, as needed with LR. Moreover, compared with traditional statistical methods, ML algorithms can handle missing data more efficiently because they do not rely on data distribution assumptions and are capable of more complex calculations. Clinical models constructed by ML have been used to predict short-term mortality in cardiac surgery with the performance regarding AUC ranging from 0.77 to 0.92 (19, 20, 39–47). Zhou et al. (39) and Ong et al. (40). Found that the RF models predict short-term mortality better than other models in cardiac surgical procedures. Additionally, several studies showed that the XGBoost method performed better in predicting operative or in-hospital mortality than the other ML methods (19, 20, 41–43). In our study, the study outcome was set as long-term mortality, and the Ada model performed better than the RF and XGBoost model. This also supports the so-called No Free-Lunch theorem in ML (48), which shows that there is no one model that works best for every problem or every dataset. Therefore, it is necessary to try and evaluate multiple ML models to determine which one performs best for a specific problem or study cohort. Actually, The Ada model is a technique that is gaining increasing application in clinical research (49–51). Our study is the first to apply the Ada model in the context of cardiac surgery.

Through sophisticated ML methods, we determined that RDW, BUN, SAPS II, AG, age, urine output, chloride, creatinine, congestive heart failure, and SOFA were the Top 10 predictors in the feature importance rankings. In general, the predictors for long-term mortality identified in the Ada model in this study are consistent with other studies. RDW is a simple measure of the broadness of erythrocyte size distribution, conventionally called anisocytosis (52). A growing body of evidence demonstrated that higher RDW is strong correlation with a higher mortality rate in widespread cardiovascular diseases such as cardiac surgery, heart failure, and acute coronary syndrome (53–56). However, there is less research available about whether RDW affects long-term outcomes after cardiac surgery, for which our study is a novel contribution to the published literature. The SAPS II, based on a large international sample of patients, provides an estimate of the risk of death without having to specify a primary diagnosis (57). According to our findings, the SAPS II score seems to be more important than the SOFA score in the feature importance rankings of the Ada model. Similar to our findings, Schoe et al. (58) found that the SOFA score used as a mortality prediction model underperformed compared to the SAPS-II score in this large cohort of cardiac surgery patients. Urine output, BUN, and creatinine were all Top 10 important variables. Lassnigg et al. (59) reported that even a slight increase in serum creatinine is correlated with a considerable increase in 30-day mortality following cardiac surgery. Tseng et al. (60) developed and validated ML algorithms using 94 preoperative and intraoperative features to predict cardiac surgery-associated acute kidney injury, which is closely associated with increased morbidity and mortality. In their model, the importance matrix plot reveals that the most important variables contributing to the model were intraoperative urine output. Our results also underline the importance of detecting, evaluating, and improving preoperative renal function in patients requiring cardiac surgery, which might serve as a target for improving outcomes.

There are several strengths of our study. Firstly, this is the first study that established advanced ML death prediction models focusing on the long-term mortality of patients undergoing all types of cardiovascular surgery. Given the heterogeneity of patients on ICU admission, our findings can be used to identify patients at high-risk for death, and determine which patients would benefit most from cardiac surgery. Providers can then offer targeted individualized care such as more extensive evaluation, post-discharge home visits, closer surveillance by primary care physician, or earlier post-operative follow-up appointments for these patients, actions that might mitigate future adverse outcomes. Secondly, we used MIMIC-III, a high-quality database with large sample size and extensive clinical data. Thirdly, we utilized advanced statistical methods, including eight ML models. To evaluate the performance of these models, the AUCs, calibration curves, and DCA were calculated and plotted, representing the discrimination, goodness of fit, and clinical application, respectively. Fourthly, the models were created based on the data readily available collected within the first 24 h after patients' admission. It is worth noting that early and accurate prediction of mortality can provide more time for clinicians to adjust corresponding treatment strategies. Finally, to help surgeons use the model at the bedside, a calculator was developed, which provided a user-friendly interface.

Our study had several limitations. Firstly, we used data from a single academic medical center in the USA, with the earliest cases from almost 20 years ago, when care may have been inconsistent with currently accepted standards. Therefore, a multicenter registry, prospective studies are needed to confirm these findings. Secondly, derived from the ICU adult participants, the results of our study cannot be generalized to other populations such as children and non-ICU patients. Thirdly, we did not obtain information including laboratory testing and interventions before ICU admission, which may cause confounders to some extent. Fourthly, restricted by the contents of the MIMIC-III database, some important information, including preoperative data (i.e., lactate, left ventricular ejection fraction, NYHA functional class, EuroSCORE score, and STS score), intraoperative data (i.e., intraoperative hypotension, vasopressor-inotropes and cardiopulmonary bypass time), and postoperative data (i.e., complications, late extubation, and length of ICU stay) were recorded incompletely and not included in the analysis. Fifthly, although we included patients in the database with the primary diagnosis of receiving cardiac surgery, it cannot be ruled out that some patients were admitted to treating other diseases. Finally, although our study deeply explored 4-year mortality in the ICU settings, other outcomes, such as acute kidney injury incidence, are also needed for further investigation.

## Conclusions

The Ada model performs better than the LR, NNET, NB, GBM, RF, BT, and XGB models in predicting long-term mortality after cardiac surgery. Our results suggest that RDW, BUN, SAPS II, AG, age, urine output, chloride, creatinine, congestive heart failure, and SOFA might be closely associated with 4-year mortality after cardiac surgery. We anticipate that this new risk model can become a handy risk stratification tool that can be used by clinicians and patients in the choice of treatment for cardiac disease. However, further external validations are warranted to test the generalization of our models.

## Data Availability Statement

The raw data supporting the conclusions of this article will be made available by the authors, without undue reservation.

## Author Contributions

YY, ZW, and ZJ conceived the analysis. YY and CP extracted all data. YY, ZZ, and KS undertook and refined the inclusion process. YY, CP, and ZW co-wrote the paper. YY, CP, YZ, and JX undertook the statistical analyses. WX, PW, and ZW were consulted for clinical issues. All authors contributed to and revised the final manuscript.

## Funding

This work was supported by the National Nature Science Foundation of China (No. 81770244), Medical Science and Technology Youth Cultivation Plan (Nos. 17QNP013 and 20QNPY038), Shanghai Municipal Commission of Science and Technology (No. 17ZR1439100), Shanghai Shenkang Medicine Developing Project (No. SHDC12014107), and Shanghai Science and Technology Committee Medicine Leading Project (No. 15411960100).

## Conflict of Interest

The authors declare that the research was conducted in the absence of any commercial or financial relationships that could be construed as a potential conflict of interest.

## Publisher's Note

All claims expressed in this article are solely those of the authors and do not necessarily represent those of their affiliated organizations, or those of the publisher, the editors and the reviewers. Any product that may be evaluated in this article, or claim that may be made by its manufacturer, is not guaranteed or endorsed by the publisher.

## Abbreviations

EuroSCORE: European System for Cardiac Operative Risk Evaluation
STS: Society of Thoracic Surgeons
ML: machine learning
MIMIC: Medical Information Mart for Intensive Care
ICU: intensive care units
CABG: coronary artery bypass grafting
SBP: systolic blood pressure
DBP: diastolic blood pressure
MBP: mean blood pressure
WBC: white blood cell
RBC: red blood cell
RDW: red blood cell distribution width
PT: prothrombin time
INR: international normalized ratio
BE: base excess
AG: anion gap
BUN: blood urea nitrogen
SOFA: Sequential Organ Failure Assessment
qSOFA: quick Sequential Organ Failure Assessment
SAPS II: Simplified Acute Physiology Score II
RRT: renal replacement therapy
ECMO: extracorporeal membrane oxygenation
IQR: interquartile ranges
RFE: recursive feature elimination
NNET: artificial neural network
NB: naïve bayes
GBM: gradient boosting machine
Ada: adapting boosting
RF: random forest
BT: bagged trees
XGB: eXtreme Gradient Boosting
LR: logistic regression
AUC: area under the curve
DCA: decision curve analysis.
