# Supplementary material for: Machine Learning Methods for Predicting Long-Term Mortality in Patients After Cardiac Surgery
Source: Front Cardiovasc Med. 2022 May 3;9:831390. doi: 10.3389/fcvm.2022.831390 (PMC9110683; doi:10.3389/fcvm.2022.831390)
Supplement: Supplementary file 1 [file Table_1.docx]

**Table S1. Missing number (%) for included variables in the dataset.**

| **Variables** | **Missing, n (%)** |
| --- | --- |
| SBP | 4.07 |
| DBP | 4.50 |
| MBP | 7.40 |
| Heat rate | 4.71 |
| Respiratory rate | 1.60 |
| Temperature | 10.80 |
| Urine output | 2.30 |
| SpO_2_ | 1.60 |
| pH | 10.96 |
| Bicarbonate | 2.41 |
| AG | 2.25 |
| BE | 13.76 |
| WBC | 5.39 |
| RBC | 1.15 |
| RDW | 6.49 |
| Hematocrit | 1.03 |
| Hemoglobin | 1.07 |
| Platelet | 4.03 |
| BUN | 7.55 |
| Glucose | 5.46 |
| Calcuim | 15.42 |
| Chloride | 2.03 |
| Creatinine | 7.55 |
| Potassium | 3.55 |
| Magnesium | 5.62 |
| Sodium | 3.25 |
| Phosphate | 16.71 |
| PT | 7.38 |
| INR | 10.64 |

*SBP, systolic blood pressure; DBP, diastolic blood pressure; MBP, mean blood pressure; WBC, white blood cell; RBC, red blood cell; RDW, red blood cell distribution width; PT, prothrombin time; INR, international normalized ratio; BE, base excess; AG, anion gap; BUN, blood urea nitrogen*
